# Case Report: Oncocytic Schneiderian Papilloma Originating From the Sphenoid Sinus

**DOI:** 10.3389/fmed.2022.621705

**Published:** 2022-04-04

**Authors:** Sergey A. Karpischenko, Olga E. Vereschagina, Olga A. Stancheva, Pavel R. Bibik, Dmitry I. Kaplun, Mikhail I. Bogachev, Airat R. Kayumov

**Affiliations:** ^1^Ear, Nose and Throat (ENT) Department, First Pavlov State Medical University, St. Petersburg, Russia; ^2^Department of Automation and Control Processes, St. Petersburg Electrotechnical University “LETI”, St. Petersburg, Russia; ^3^Research Centre for Digital Telecommunication Technologies, St. Petersburg Electrotechnical University “LETI”, St. Petersburg, Russia; ^4^Institute of Fundamental Medicine and Biology, Kazan Federal University, Kazan, Russia

**Keywords:** oncocytic Schneiderian papilloma, sphenoid sinus, FESS, CT scan examination, nasal cavity, paranasal sinuses

## Abstract

A rare case of oncocytic Schneiderian papilloma originating from the sphenoid sinus characterised, for 3 years, by non-specific symptoms of severe headache, a block of nasal breathing, and deprecating sense of smell was presented by an elderly female patient. Sphenoid sinus functional endoscopic sinus surgery (FESS), with a one-block tumour excision, through an endonasal approach, with a histological study of removed tumour masses, were performed on the patient. Long observation in the post-operative period was necessary, considering the risk of recurrence and malignancy of oncocytic Schneiderian papilloma (OSP). Although the oncocytic papilloma of the sphenoid sinus is rare, non-specific symptoms make this pathology easily misdiagnosed. Thus, any isolated unilateral process in the paranasal sinuses with long-existing symptoms must be given careful attention due to the chance of this process being an inverted papilloma with malignization. CT scan indicating a unilateral opacification of paranasal sinuses with local calcifications is a typical manifestation, and endoscopic sphenoidotomy can be recommended as a treatment of choice.

## Introduction

Oncocytic Schneiderian papilloma, also known as cylindrical cell papilloma, is a rather uncommon type of epithelial tumour that develops from the Schneider's membrane. The Schneiderian membrane comprises a few layers, including the epithelial lining, the lamina propria, and the bone interface. This membrane includes a richly vascularized lamina propria; osteoprogenitor cells which may be associated with cells, pericytes, within the microvascular walls (1) or in the bone marrow as adventitial subendothelial cells (2). Exophytic inverted papilloma and oncocytic Schneiderian papilloma (OSP) ordinarily arise from the lateral part of the nasal cavity (3). Arising from the maxillary sinus, nasal cavity, and ethmoidal sinus were the most frequently found, whereas its exclusive location within the frontal or sphenoid sinus was exceedingly rare (only 5% of all paranasal sinuses) (4, 5). Schneiderian Papillomas can also originate outside the synonasal tract. They can be found in the middle ear cavity and the mastoid, nasopharynx, pharynx, and lacrimal sac (6). There are rare, as a group and represent only.4–4.7% of all neoplasms if the nasal cavity and paranasal sinuses (7), while OSP, the rarest subtype, occurs in 3–5% if cases (up to 13 % of cases, by other sources) (8). During the development of OSP, there was no sex preference (9). At the time of the diagnosis, the majority of the patients were above 50 years old. The youngest patient with OSP, reported in the literature, was a 33-year-old woman (10). It is important to note that between 4 and 17% of the total OSP have been associated with malignancy (11).

Unilateral lesions of the sphenoid sinus should be differentiated from other inflammatory pathologies in the sphenoid sinus or tumours. Lawson et al., in their analysis of isolated sphenoid sinus disease, found 10 benign and 18 malignant tumours out of 132 cases (12). According to the literature, inverted papilloma is the most common benign tumour of the sphenoid sinus (13). Other benign such as hemangioma, fibro-osteoma, schwannoma, pseudotumour, plasmacytoma, myxofibroma, and osteochondroma have cinoma, but the latter contains only monolayered epithelium (14). For inflammatory changes in the sphenoid sinus, a bilateral opacification with the lesion or the involvement of neighbouring structures (posterior ethmoid cells) is usually presented. Such changes are identified on CT scans as pathological masses with smooth and clear contours with liquid components.

The most common clinical presentation of IP is unilateral nasal obstruction and epistaxis, such symptoms that arise as a result of lateral nasal wall involvement (15–17). However, patients with inverting papillomas confined to the sphenoid sinus often manifest with occipital/frontal headaches or retro-orbital pain radiating downward towards the neck. Subsequent expansion of the sinus walls then results in compromised cranial nerve function and visual field defects (5). For isolated sphenoid sinus inverted papilloma besides the headache is also epistaxis and eye movement problems are common as reported in (13) based on four patient's observations.

Ethiology of Schneiderian Papillomas is still unclear. To date, the roles of the mucosal membrane allergic reactions, chronic sinusitis, air pollution, and viral infections are unclear. The etiopathogenetic factors of either inverted or fungiform Papillomas development include human Papillomavirus (HPV), mainly of the types 6, 11, 16, and 18. However, there is no official supporting data that HPV is a provocative factor in OSP development (18, 19). OSP appears as a sift, fleshy pink papillary tissue. Histologically, it exhibits both the exophytic and the endophytic type if growth, containing 2–8 layers if respiratory epithelium cells, also including small dark round nuclei, eosinophilic cytoplasm, and reduced epidermoid component. Similarly, to the inverted Papilloma, numerous small cysts, containing intraepithelial mucus or neutrophils, can be observed within the epithelium. It was shown previously that the cells do not only resemble oncocytes but are also characterised by high levels of cytochrome C oxidase while being ultrastructurally extended with mitochondria, thus obviously establishing their oncocytic characteristic. In a few of the outermost cells, in varying stages of regression, cilia can also be observed (20). If a unilateral process in the nasal cavity and the paranasal sinuses on the CT scan is discovered, then the patient needs to be evaluated for the presence of neoplasm formations. By using CT scanning with contrast fluid, foci of calcification can be visualised, leading to a benign characteristic of the process. Calcifications can be represented by dot-shaped, linear, or circular inclusions (21). MRI is invariably complementary to CT due to the superior soft-tissue contrast multiparametric resolution. MRI can help define the typical inverted papilloma cerebriform pattern, the extent of pathological contrast uptake, and the presence of focal alteration (22). The treatment consists of the endoscopic resection of the entire tumour with the mucosa. In some cases, the drilling of the tumour growth zone is necessary to prevent a recurrence, once it was identified during the surgery. During surgery, the tumour tissue and surrounding mucosa should be removed together to prevent post-operative recurrence better. The bone hyperplasia in the basal part of the tumour needs to be removed with a bone chisel or drill (23, 24). If the drilling is not performed in the correct zone, at least 25–35% of the patients exhibit a recurrence, usually within 5 years after surgery (25).

## Case Report

A patient who is a 85-year-old woman of Caucasian descent complained of a severe headache, a block of nasal breathing, and a decreased sense of smell for 3 years. The patient reported hypertension in the medical history, without additional related hereditary diseases, also in the family history. No previous surgical interventions of the nasal cavity or paranasal sinuses have been reported.

**Figure 1:**
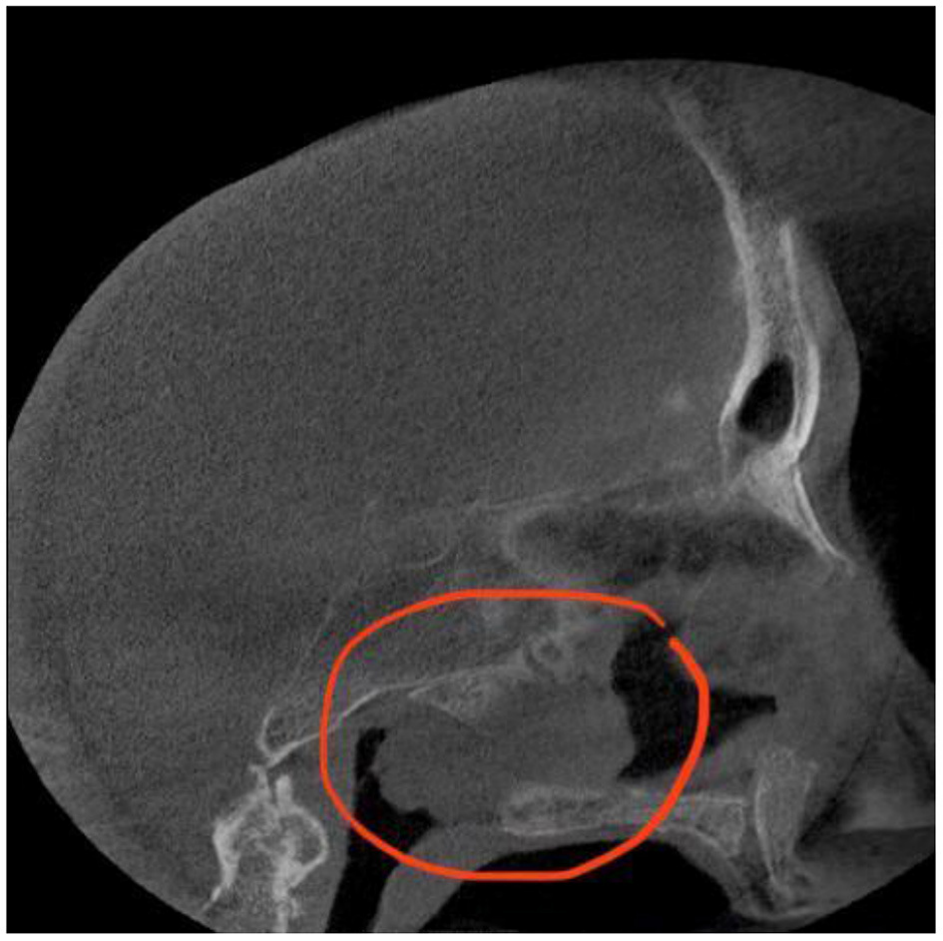
Pre-operative CT scan sagittal view.

First, she was observed by an ear, nose, and throat (ENT) specialist and then diagnosed with chronic isolated sphenoid sinusitis. Since topical corticosteroid therapy and oral antibiotics (2 g of Amoxicillin/Clavulanic Acid 875 mg/125 mg every 12 h for 7 days) were not effective, the patient then was submitted to a CT scan examination (Figure 1).

Considering the suspicion of a tumour and the absence of bone destruction of the sphenoid sinus, it was decided on a wide endoscopic resection of the lesion in one block. Intraoperative biopsy confirmed the diagnosis of OSP, and the bone drilling at the attachment site was performed (26).

Radiation therapy was not recommended for this patient due to tumour location (27).

The observation period for such patients should be at least 5 years since inverted papilloma is characterised by a high recurrence rate (14, 28).

In the follow-up period, an otolaryngologist observed the patient in her city. After 5 years, she was re-invited for an endoscopic control examination and provided a CT scan conclusion. All symptoms, including the headache and block of nasal breathing, were reduced.

The most important point is drilling out of the tumour matrix from the bone and subsequent careful observation of the intervention area.

A lesion was found to be an opacification that was expanding into the right sphenoid sinus, extending to the nasal cavity on the unilateral side. The radiologist described this tumour as a lobulated homogeneous mass. The sinus wall was thinned, but there were no signs of intracranial invasion or defects of bony borders of the sinus. The anterior sphenoid sinus wall was detected to be the tumour matrix.

**Figure 2:**
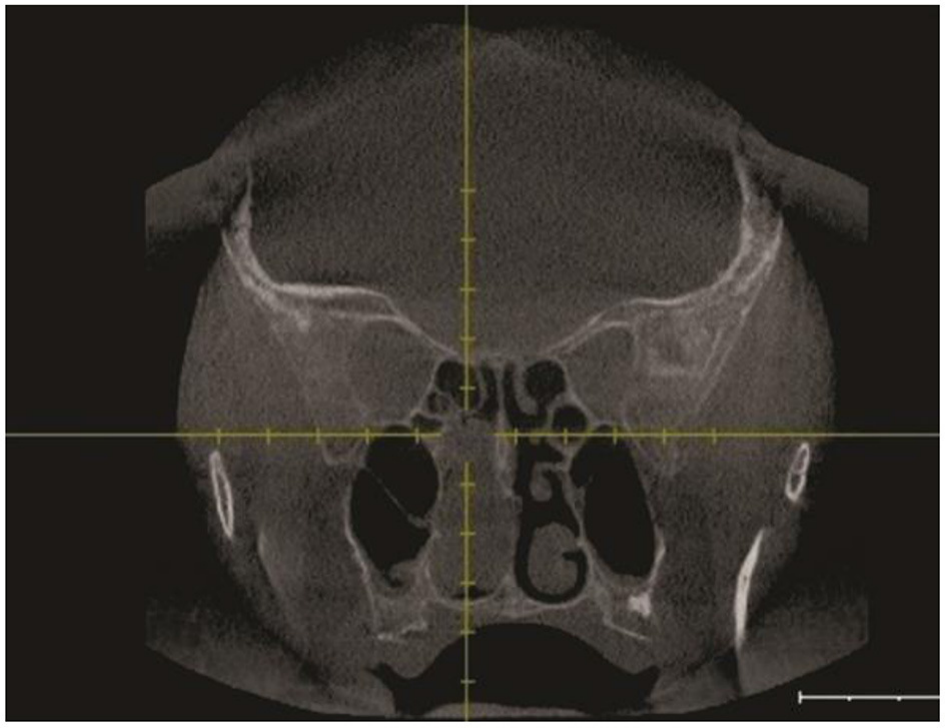
Pre-operative CT scan coronal view.

Following the CT scan, endonasal endoscopic imaging was performed. Sinus CT (coronal view) (Figure 2) demonstrates a soft tissue mass that completely opacified the right sphenoid sinus extending to the nasal cavity. Sagittal view of head CT showed that neither the carotid artery nor the optic nerve was involved in the pathological process.

**Figure 3:**
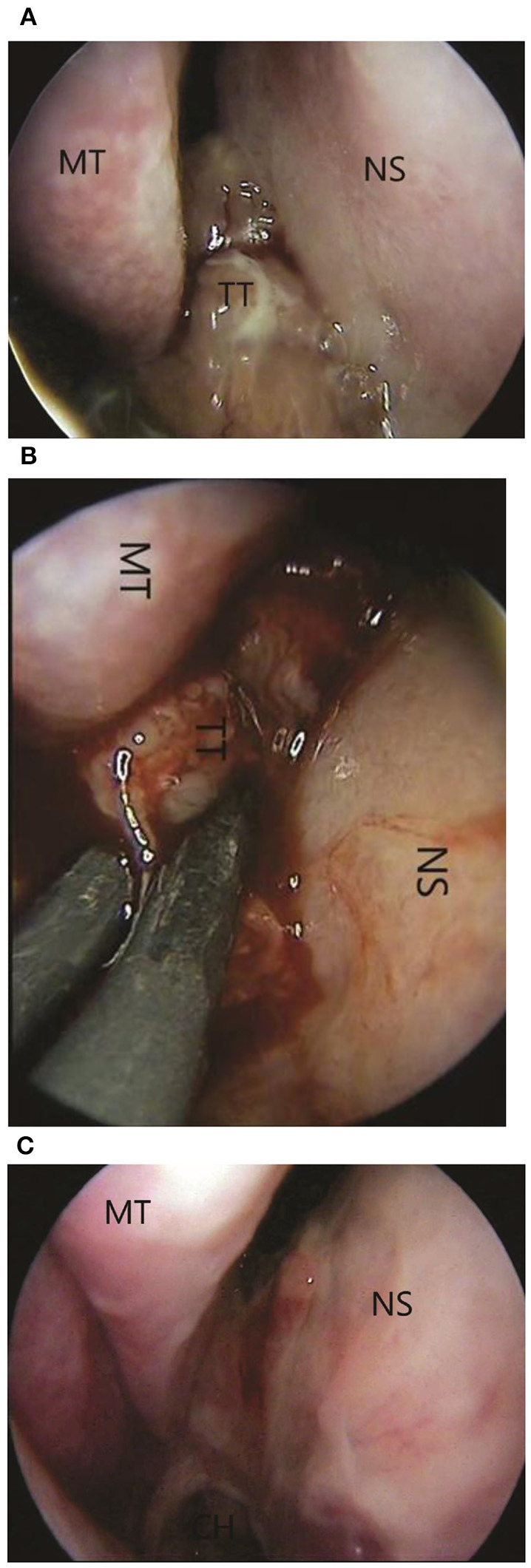
**(A)** Pre-operative 0-degree endoscopic view of tumour: MT, middle turbinate; NS, nasal septal wall; TT, tumour tissue. **(B)** The intraoperative view. The using of coagulation: MT, middle turbinate; NS, nasal septal wall; TT, tumour tissue. **(C)** One-month postoperative 0-degree endoscopic view. No signs of recurrence.

**Figure 4:**
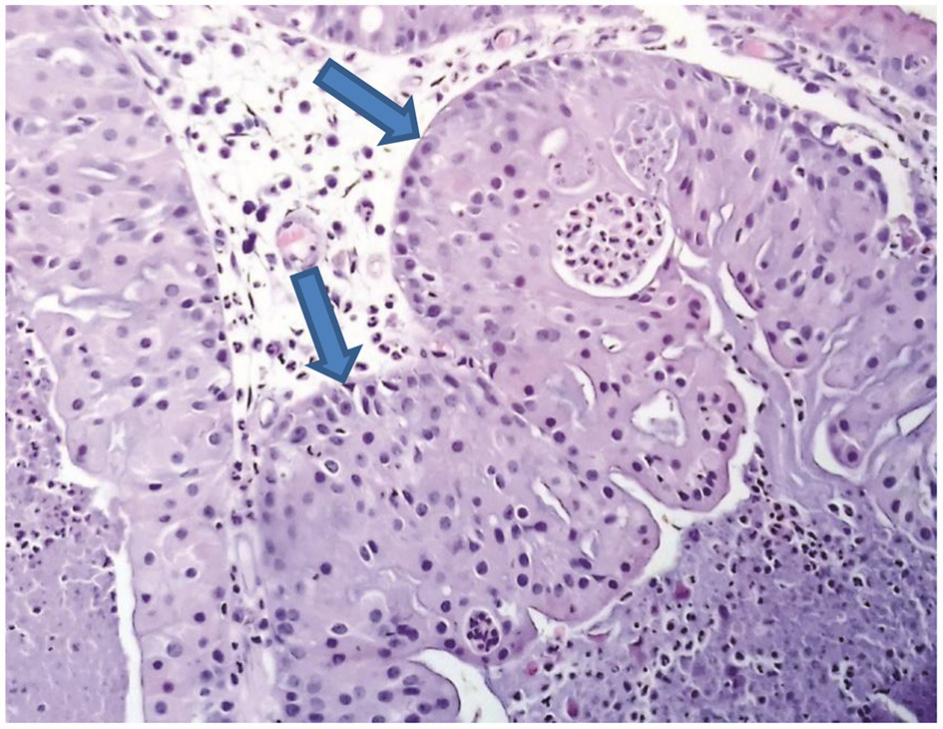
H & E staining. Structures of oncocytic Schneiderian papilloma.

Next, the patient was subjected to a sphenoidotomy by 0- or 30-degree rigid endoscope under general anaesthesia (Figures 3A,B). The main part of the tumour was removed by microdebridor. Additionally, tumour margins were identified and widely resected. There was no intensive bleeding during the operation. The following biopsy confirmed the diagnosis of the oncocytic Schneiderian tumour (Figure 4). The patient showed positive signs of recovery and was discharged after 5 days post-operatively. Endoscopy of the nasal cavity was performed with no signs of recurrence in the 5-year follow-up period (Figure 3C).

### Objectives

Clinical manifestations, diagnostic procedures, and treatment of a rare case of OSP originating from the sphenoid sinus are reported.

### Methods

Analysis of symptoms, localisation, pre-operative OT scans, and surgery performed on the patient with OSP originating from sphenoid sinus, followed by a follow-up period.

## Results

Non-specific symptoms of severe headache, a block of nasal breathing, and deprecating sense of smell, over a period of 3 years, were presented by an elderly female patient. Sphenoid sinus functional endoscopic sinus surgery (FESS), with a block tumour excision, through an endonasal approach, with a histological study of removed tumour masses, was performed on the patient. Long observation in the post-operative period was necessary, considering the risk of recurrence and malignancy of OSP.

## Discussion

The oncocytic Papilloma (OP), also identified as columnal cell papilloma, is an uncommon benign neoplasm growing from the Schneiderian membrane which occurs only in 3 to 5% out of the three types mentioned above of Papillomas cases. The ectodermal-derived mucosa of the nose and paranasal sinuses, the so-called Schneiderian membrane, may give rise to three morphologically distinct papillomas, excluding the keratinizing squamous papilloma of the nasal vestibule. These are referred to individually as the fungiform (exophytic, septal, squamous), inverted, and cylindrical cell papillomas (CCPs) or, collectively, as Schneiderian papillomas (14). It usually arises from the maxillary sinus or ethmoid cells, while more rarely, from the frontal sinus and the sphenoid sinus, respectively. It is characterised by aggressive local growth and significant malignant potential. Usually, OP can be identified in the 4th and the 5th-decade of life.

A feature of this article is a rare case of Schneiderian papilloma in geriatric patients. The patient's main complaint was the block of nasal breathing without any headache, ophthalmological complications, or postnasal drip, which are typical for sphenoid sinus opacification. It is very important to identify the pathology in the early stages. Clinical manifestations are largely reminiscent of the primary acute sinusitis, while often exhibiting unilateral character and resembling polyposis masses in the endoscopic view. Clinical manifestations of nasal cavity diseases are reduced in geriatric patients.

The latest research into the diagnosis of inverted papilloma with the Storz Professional Image Enhancement System (SPIES) and White Light Endoscopy (WLE) showed a high sensitivity of the former, confirmed by histological data (29). Such a minimally invasive method will be very useful in patients with a poor clinical picture, such as geriatric patients.

Another additional factor in the diagnosis of inverted papillomas is the titer of antibodies to human papillomavirus. Studies note that high levels of human papillomavirus are directly correlated with inverted papilloma of the nasal cavity and sinuses. As noted in the study, cell proliferation was found much more frequently on histopathological examination of inverted papilloma compared to the study of polyps. The principal hypothesis regarding the ability of HPV to induce cellular proliferation in various mucosa lesions is based on the fact that the virus uses the replication system of the host cell, as the virus itself does not code for replication enzymes (30).

In some cases, inverted papillomas appear reminiscent of polyp-like masses in combination with chronic sinusitis in the CT scan images. Inverted papilloma of the sphenoid sinus is caused by the development of serious complications due to the location of the sinus. Therefore, it is doubly difficult to detect sinus masses in these patients. Thus, we recommend that all patients over 70 perform CT of the sinuses and endoscopy if they have complaints of nasal congestion. Papillomas always affect one side of the nasal cavity leading to the dilatation of the sinus walls, followed by bone erosion. An intracranial extension might happen; however, it is always an indicator of malignancy disorders of the mucosa. The treatment of choice is usually a complete removal of the lesion with wide resection, including the removal of the normal margins of the mucosa. It was identified that the removal of the mucosa from the affected sinus leads to a better outcome in the follow-up period, indicated by the fewer case of recurrence. The endoscopic approach appeared to be more effective, especially in the initial stages of the disease, with an opacification of one or two parts of the nasal cavity. The radiation therapy had been reserved in the cases of inverted Papilloma in association with squamous cell carcinoma. Other reports indicate that neither chemotherapy nor radiation therapy is effective.

## Conclusion

Any isolated unilateral process in the paranasal sinuses, with long-existing symptoms, must be given careful attention due to the chance of this process being an inverted papilloma with malignization, therefore a histological confirmation must be completed.

Although the OP of the sphenoid sinus is rare, non-specific symptoms make this pathology easily misdiagnosed. The CT scan indicating a unilateral opacification of paranasal sinuses with local calcifications is a typical manifestation, and endoscopic sphenoidotomy can be recommended as a treatment of choice (31, 32).

## Data Availability Statement

The original contributions presented in the study are included in the article/supplementary material, further inquiries can be directed to the corresponding authors.

## Ethics Statement

Ethical review and approval was not required for the study on human participants in accordance with the local legislation and institutional requirements. Written informed consent for participation was not required for this study in accordance with the national legislation and the institutional requirements. Written informed consent was obtained from the individual(s) for the publication of any potentially identifiable images or data included in this article.

## Author Contributions

SK, OV, OS, PB, DK, MB, and AK contributed to the concept and design, acquisition, analysis and interpretation of the data, drafting and critical revision of the article, and giving final approval. All authors contributed to the article and approved the submitted version.

## Funding

The reported study was funded by the Russian Science Foundation, project number 21-79-20219.

## Conflict of Interest

The authors declare that the research was conducted in the absence of any commercial or financial relationships that could be construed as a potential conflict of interest.

## Publisher's Note

All claims expressed in this article are solely those of the authors and do not necessarily represent those of their affiliated organizations, or those of the publisher, the editors and the reviewers. Any product that may be evaluated in this article, or claim that may be made by its manufacturer, is not guaranteed or endorsed by the publisher.
